# Active Coping and Anxiety Symptoms during the COVID-19 Pandemic in Spanish Adults

**DOI:** 10.3390/ijerph18168240

**Published:** 2021-08-04

**Authors:** Raquel Lara, Martha Fernández-Daza, Sara Zabarain-Cogollo, María Angustias Olivencia-Carrión, Manuel Jiménez-Torres, María Demelza Olivencia-Carrión, Adelaida Ogallar-Blanco, Débora Godoy-Izquierdo

**Affiliations:** 1 Departamento Psicología Social, Facultad de Psicología, Universidad de Granada, 18071 Granada, Spain; 2 Grupo de Investigación Psicología de la Salud/Medicina Conductual (CTS-267), Universidad de Granada, 18071 Granada, Spain; maolivencia@ugr.es (M.A.O.-C.); mjitor@ugr.es (M.J.-T.); adelaidaogallar@ugr.es (A.O.-B.); 3 Psychology Department, Universidad Cooperativa de Colombia, Santa Marta 110000, Colombia; martha.fernandezd@campusucc.edu.co (M.F.-D.); sara.zabarain@campusucc.edu.co (S.Z.-C.); 4 Grupo de Investigación Estudios Sociales Interdisciplinares-ESI, Santa Marta 110000, Colombia; 5 Departamento de Personalidad, Evaluación y Tratamiento Psicológico, Facultad de Psicología, Universidad de Granada, 18071 Granada, Spain; 6 Centro de Salud de Torredonjimeno, 23000 Jaén, Spain; dolca80@hotmail.com

**Keywords:** anxiety, coping, exercise, COVID-19, mental health

## Abstract

The features of the COVID-19 pandemic and the social operations to contain the spread of the virus might have limited or altered coping, including healthy habits such as exercise, this contributing to a myriad of negative consequences for the mental health of the global population. We explored the contribution of coping and physical activity to the management of anxiety in Spanish adults during an active phase of the epidemic, as well as the relationship between these strategies. A total of 200 young and adult individuals (70% women) voluntarily completed an anxiety inventory, a coping skills self-report and a personal data section including exercise practice. The participants reported in average a mild yet existing level of anxiety symptoms; a third reported noticeable symptoms. At the time of the study, the participants used more adaptive than maladaptive coping styles. Participants’ anxiety was inversely correlated with an active coping style, and positively with an avoidant style; physical activity correlated positively with an active coping style, and regular exercisers used more frequently active coping. Controlling for confounders, active coping, avoidant coping and exercise during the pandemic predicted anxiety symptoms. Other findings indicated that exercise was used as a coping strategy for dealing with emotional distress. Our results highlight the positive impact of functional coping and exercise for the management of negative states such as anxiety during the pandemic, and underline the importance of developing interventions aimed at enhancing coping skills for promoting physical and mental well-being of the population during health and social crises.

## 1. Introduction

The World Health Organization (WHO) declared on 12 March 2020 that the new 2019 coronavirus (SARS-CoV-2) and its related syndrome (COVID-19) constituted an official pandemic due to the 125,000 cases detected in 118 countries at that moment [1]. The COVID-19 pandemic has spread rapidly worldwide and, given the absence of effective vaccines until very recently, strict controlling measures (e.g., quarantines; movement restrictions, stopping mass gathering and social isolation; risk-reducing personal actions) differing in duration and severity have been the only possible interventions to protect peoples’ health. Despite the fact that the effectiveness of these procedures has been well established [2,3], their outcomes in terms of mental health are still unclear. Several investigations [2,3,4,5,6,7] and reviews of previous outbreaks [8,9] stress the consequences of the recent COVID-19 outbreak and related quarantine actions in terms of a variety of psychopathological manifestations.

Fear of infection, social distancing and separation from loved ones, loss of freedom, prohibition of common activities (e.g., exercise-sporting activities) and the closure of recreational facilities (parks, gyms, cinemas, theaters) can make coping quite complicated. In addition, other difficulties such as changes in daily life, economic and job problems or, on the contrary, exigent demands in terms of work overload and family conciliation, could also challenge the adoption of coping strategies [2,4,10]. Emotional distress (e.g., sadness, worry, loneliness, stress, hypervigilance, insomnia) should be considered a normal way in which people react to uncertain and dangerous situations, as a global pandemic. Nevertheless, an increased incidence of psychopathological disorders (e.g., stress, anxiety, depression, post-traumatic stress) has been documented in several systematic reviews [3,4,11,12,13,14] and meta-analysis [15,16]. Specifically, a study [17] found that, of all participants in the reviewed studies, 20.8% had clinically meaningful levels of psychological distress, 22.7% of depressive symptoms, 21.7% of post-traumatic stress and 16.2% of anxiety symptoms during and after quarantine periods. In addition, it has been shown that the first weeks after the pandemic started in China there was an increase in negative emotions (anxiety and depression) but also a decrease in life satisfaction [18].

Research has also supported that the adverse effects of COVID-19 pandemic in terms of psychological distress are largely derived from dysfunctional cognitive, behavioral and emotional coping efforts [19,20]. Behavioral, cognitive and emotional coping strategies have been found to be helpful for dealing with negative experiences, since they reduce the burden imposed by prolonged distress, enable cognitive resources to deal with everyday stressors and allow adaptation to environmental changing and demanding situations during pandemics and natural disasters [21,22]. Furthermore, coping has been demonstrated to be a key factor for psychological well-being and mental health issues during the COVID-19 era, with problem-focused coping strategies, cognitive reappraisal, social support and avoidant coping strategies along with other coping resources such as meaning in life and social connectedness as the most commonly used coping strategies [19,20,23,24,25,26,27,28,29,30]. A review [31] has found that all the guidelines for coping with mental problems derived from COVID-19 included tips for maintaining good mental health, descriptions of a variety of psychological skills to help people cope with their anxiety and worries and the promotion of interpersonal connection at home to generate social support. Nevertheless, the role of coping for emotional distress during this crisis has been scarcely investigated.

On the other hand, the imposed restrictions during the different quarantine periods have limited people’s physical activities, which has led to an increase in sedentary lifestyle in the population [32,33,34,35]. All but one of the studies included in a review [35] reported a decrease in the volume of physical activity during or after COVID-19-related confinements compared to pre-pandemic levels. These data are congruent with other investigations [36,37,38,39,40,41] reporting that more than 50% of the examined population decreased their exercise practice during confinement.

This reduction in exercise has a deleterious effect on physical, mental and social health [32,42,43,44,45,46,47]. The decrease in physical activity is associated with higher negative affect and anxiety and lower levels of energy [48,49,50] as well as to the worsening of other health-related behaviors (e.g., diet, tobacco and alcohol consumption, sleep behaviours) during confinement [46,51]. On the contrary, staying active or becoming active during the pandemic reduces the risk of depression and anxiety, predicts recovery experiences and improves emotional well-being and life satisfaction [52,53,54,55,56,57,58]. Consequently, physical activity is considered a protective factor [59,60]. Several reviews support the relationship between exercise during the COVID-19 pandemic and better mental health [61,62,63]. The practice of exercise along with spending time with family, talking with friends, healthy eating, sleep hygiene and investing time in pleasant hobbies have been considered successful strategies for coping with mental distress during COVID-19, these practices being associated with better psychological health [64,65,66,67,68]. This has led some researchers to consider the practice of physical activity as a coping strategy itself [69].

The WHO [1] has recognized the importance of paying attention to the mental health of the general population during the COVID-19 pandemic. Understanding how effective coping strategies during the pandemic operate is important, not only to gain knowledge on their health outcomes but also to help developing public health interventions in specific populations, such as the promotion of functional coping strategies for restrictive quarantine situations [70].

Therefore, the main aim of this study was to examine anxiety manifestations and coping strategies adopted to manage anxiety, including the practice of physical activity, during an active stage of the COVID-19 pandemic in Spain (December 2020–February 2021, which corresponded to the period between the ending of the second wave and the beginning and peak of the third wave of the pandemic in this nation, according to the Ministry of Health of the Government of Spain) in adults of both sexes with different personal and sociodemographic characteristics. We hypothesized that anxiety symptoms would be noticeable (hypothesis 1) and also that the management of anxiety would be executed through both functional and dysfunctional coping (hypothesis 2). We also hypothesized that levels of physical activity would be lower compared to periods before the beginning of the pandemic (hypothesis 3), as well as that it would be a positive resource for anxiety management (hypothesis 4).

In addition, the present study also has the following specific objectives: (1) To determine if specific coping strategies and styles are associated with different anxiety levels. We expected to find that functional coping skills would be associated with lower anxiety, and that more dysfunctional coping would be associated with increased anxiety (specific hypothesis 2.1). We also expected that exercise practice would be linked to the use of other functional coping skills (specific hypothesis 4.1) and to decreased anxiety manifestations (specific hypothesis 4.2). (2) To determine whether participants with different levels of physical activity during the COVID-19 pandemic show differences in their efforts for coping with mental distress. We hypothesized that those participants who regularly practiced exercise would use more functional coping styles, and that non-practitioners would endorse more dysfunctional coping styles (specific hypothesis 4.3). (3) To explore the impact of coping styles and levels of physical activity on the participants’ anxiety manifestations. We hypothesized that more functional coping styles would predict lower anxiety symptoms, compared to dysfunctional styles (specific hypothesis 2.2); furthermore, exercise would also have a positive impact on anxiety reducing symptoms of emotional distress (specific hypothesis 4.4).

## 2. Materials and Methods

### 2.1. Participants

Two hundred Spanish individuals of both sexes (70% women) with ages between 18 and 74 years and different personal and sociodemographic conditions (Table 1) voluntarily participated in this study. This was a non-probabilistic convenience sample. The sample size was estimated prior to the study using the Clinical and Translational Science Institute (University of California, San Francisco, CA, USA) online calculator for clinical correlational research [71] in 194 participants for an α of 0.05, a β of 0.02, and expected *r*s for associations among the study variables previously reported (e.g., *r* coping-mental health indicators≈ 0.20 [20]).

Of them, 8% had shown symptoms of COVID-19 or were diagnosed as positive for infection at the time of the study (December 2020–February 2021). According to the Ministry of Health of the Government of Spain, it is estimated that in 1 March 2021, about 3,204,531 out of 47,351,567 Spanish individuals had been diagnosed with COVID-19, corresponding to a 6.7% of the population; thus, the percentage of participants in the present study affected by the infection fits the national proportions of the pandemic for that moment. On the other hand, 86.5% reported being sedentary on a regular basis at the time of the pandemic, compared to 20.5% reporting being sedentary before the pandemic.

### 2.2. Measures

**Figure 1:**
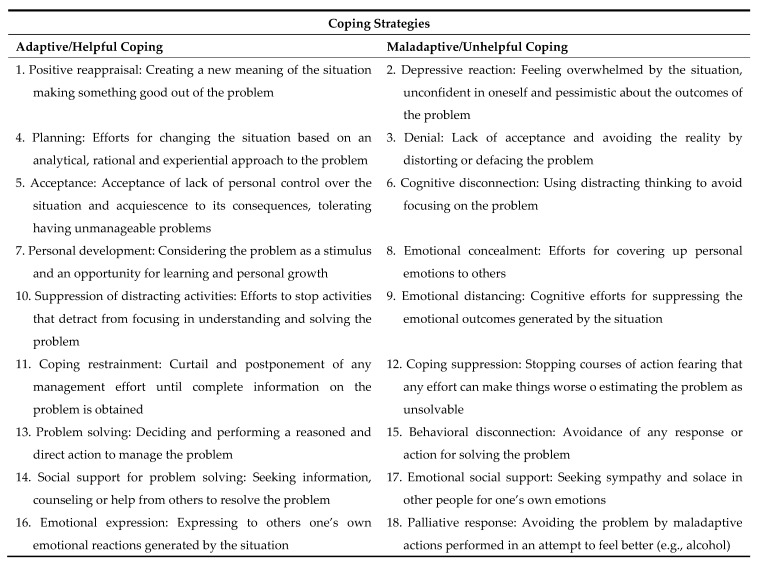

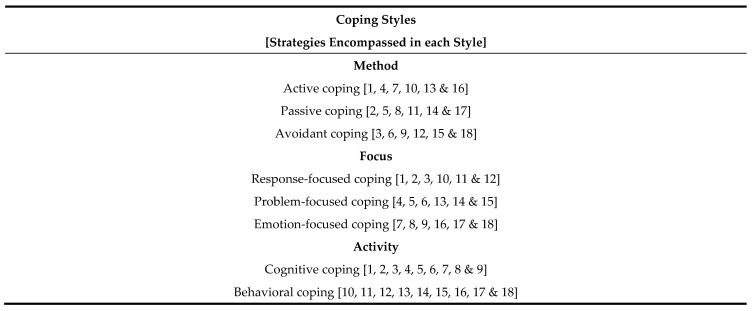
Coping strategies and styles.

The following measures were used:

- *Zung’s Self-Reported Anxiety Scale* (SAS), Spanish version [72]. The SAS is a 20-item self-report measure designed to assess levels of psychological (*“I feel scared for no reason”*, *“I feel like I’m falling and breaking apart”*) and somatic (*“My arms and legs are shaking”*, *“I feel my heart beating fast”*) symptoms of anxiety through four types of manifestations during the last week: cognitive, autonomic, motor and central nervous system symptoms. Each item is scored on a Likert-type scale from 1 (never) to 4 (most of the time). Some questions are worded negatively to avoid response bias. Total raw scores range from 20 to 80 points. This raw score is converted to an “Anxiety Index” using a provided conversion table. According to this Anxiety Index, four levels of anxiety are differentiated: absence of anxiety (20–44 points), mild anxiety (45–59 points), moderate/severe anxiety (60–74 points) and extreme anxiety (≥75 points). The SAS has well-established psychometric properties [73,74] and has been widely used, also in studies conducted during the COVID-19 pandemic [75,76]. The Spanish validation studies of the SAS reported a Cronbach’s α value of 0.88 [77]. In the present study, α was 0.84.

- *Scale of Styles and Coping Strategies E^3^A* [78]. This 72-item self-report evaluates 18 coping strategies (with four items for each one of the strategies): positive reappraisal, depressive reaction, denial, planning, acceptance, cognitive disconnection, personal development, emotional concealment, emotional distancing, suppression of distracting activities, coping restrainment, coping suppression, problem solving, social support for problem solving, behavioral disconnection, emotional collapsed in each of the three categories of styles; the interpretation of each of the coping expression, emotional social support and palliative response; and eight different coping styles, based on the methods, focus and type of activity used: active, passive and avoidant coping; response-, problem- and emotion-focused coping; and behavioral and cognitive coping (see Figure 1). Each item is rated using a Likert-type scale from 0 (never) to 3 (always). The inventory correction procedure has two phases: correction of coping strategies and correction of coping styles. First, the 18 coping strategies are scored; the higher the score obtained, the more commonly that coping strategy is used. Secondly, coping styles are scored by adding the scores obtained in the corresponding coping strategies, noting that each strategy in styles is the same as that of the coping strategies. The authors reported a Cronbach’s α of 0.73 for the complete scale, with subdimensions’ and styles’ α up to 0.73 and 0.83, respectively [79]. In the present study, α was 0.90.

In addition, participants answered questions about their sociodemographic and personal data (age, sex/gender, marital status, educational level, employment status, number of children, number of cohabitants). They also self-reported their level of physical activity before and during the pandemic on a weekly-time spent, on a scale where 0 = nothing, 1 = 30 min/week as much, 2 = 1 h/week as much and 3 = 2 h or more/week. We decided to use a global “duration” index, instead of other indicators (e.g., intensity, frequency) given that these other features of practice might be limited by the imposed restrictions. This decision is based on the fact that even moderate levels of physical activity are related to anxiety reductions [80]. Based on their responses, the participants were classified as sedentary (response 0), mildly active (responses 1 and 2) and sufficiently active (response 3). Finally, they indicated if they had had symptoms of COVID-19, whether or not they had been diagnosed with COVID-19 by means of a positive test, or if they had not had symptoms or a diagnosis of the disease.

### 2.3. Procedure

Both the recruitment of participants and the administration of the measures were carried out online. First, we presented the study to the community and requested collaboration through different social resources (e.g., Facebook, Whatsapp, Instagram, email) intending to reach a wide and heterogeneous sample. People who decided to collaborate were directed to an online survey carried out through Google Forms where detailed information about the study, its aims and the rights of the participants were presented. They then confirmed a consent blank and received specific instructions on how to complete the measurements before starting the survey. All measurements were completed in a single application by the participants. Finally, the database was downloaded and checked. Participants not meeting inclusion criteria (i.e., being 18 yr. or older, Spanish nationality or residence >1 year, reading and writing Spanish fluently, voluntary participation) or with exclusion criteria (i.e., severe physical or mental health issues) were removed from the analyses. Given that all questions in the survey were mandatory, there were no participants with missing or incomplete data who were removed from the analyses.

### 2.4. Study Design and Data Analyses

This is a correlational cross-sectional study. Preliminary and exploratory analyses of the data were conducted to ensure the adequacy of the input and to check missing data or outliers, as well as to verify parametric assumptions. Although the normality test indicated that most of the variables did not fit a normal distribution (Kolmogorov-Smirnov’s test, *p* < 0.05), the Levene’s test confirmed the homogeneity of the variances for most variables (*p* > 0.05), and thus we performed parametric tests for the statistical analyses. Besides descriptive analyses, we conducted Pearson’s *r* correlations, Student’s *t* tests, one-way ANOVA and post hoc comparisons (Bonferroni’s or Games-Howell’s tests, when appropriate) and hierarchical multiple linear regressions. The significance level for all tests was established at *p* < 0.05.

## 3. Results

Table 2 shows the descriptive results for participants’ anxiety symptoms and coping strategies and styles. The mean (raw) level of anxiety at the time of the study was 42.4 (*SD* = 9.5). According to the Anxiety Index, participants were classified into the categories of: absence of anxiety (*n* = 122, 61%), mild anxiety (*n* = 65, 32.5%) and moderate-severe anxiety (*n* = 13, 6.5%); none of the participants was classified as having extreme anxiety. In addition, of the 18 coping strategies assessed, the most used were: Personal development, Acceptance, Positive reappraisal, Problem solving, Planning and Emotional social support. Among the coping styles, the most used was the Active method.

In addition, Table 2 shows the significant correlations among anxiety, coping strategies and styles and exercise practice (during the study). Participants’ anxiety inversely correlated with four of the 18 coping strategies, namely Positive reappraisal, Acceptance, Personal development and Problem solving and one of the eight coping styles, i.e., Active coping; it was positively associated with five strategies, namely Denial, Cognitive disconnection, Emotional distancing, Social support for problem solving and Palliative response and one coping style, i.e., Avoidant coping. In turn, exercise practice was positively associated with the use of three coping strategies, namely Planning, Personal development and Problem solving and one coping style, i.e., Active coping. The zero-order association between anxiety symptoms and physical activity was non-significant (*p* = 0.349).

**Figure 2:**
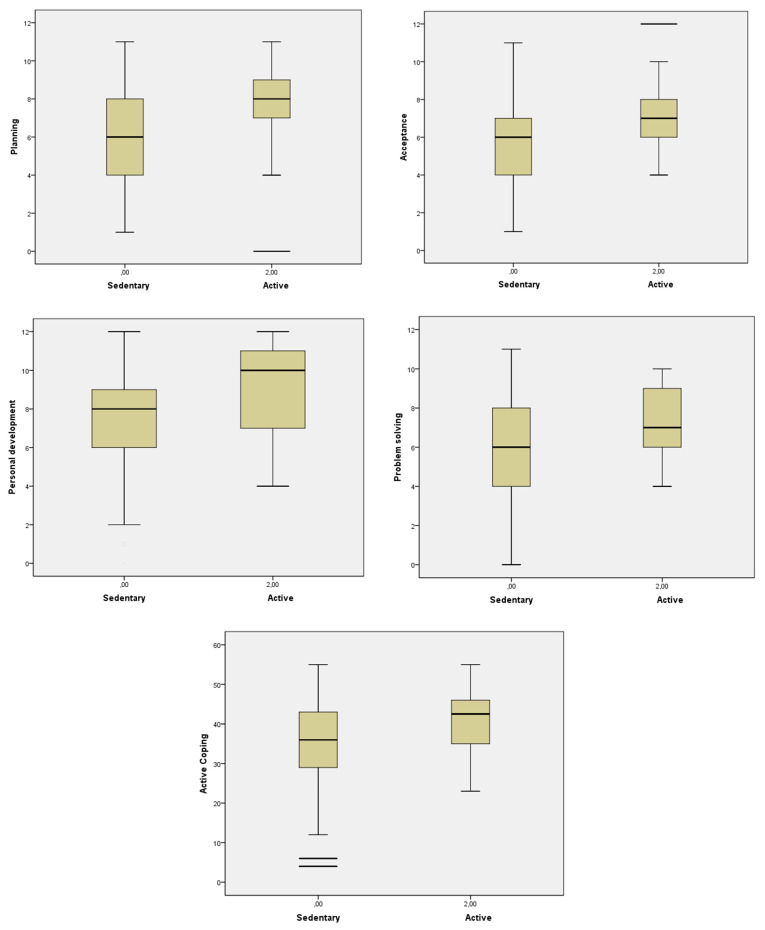
Comparisons of coping strategies between the different levels of physical exercise practice: SA (sedentary) vs. AA (active).

Table 3 shows means and standard deviations for the 18 coping strategies and eight coping styles evaluated by levels of physical activity practice. According to their global scores, the participants were classified into three levels of exercise practice during the pandemic: sedentary (SA) (*n* = 173, 86.5%), slightly active (LA) (*n* = 5, 2.5%) and active (AA) (*n* = 22, 11%). These three subgroups reported significantly different levels of practice (*F* = 3735.491, *p* < 0.001), with significant differences for coping style, i.e., Active method, and four of the 18 coping strategies (Planning, Acceptance each pair comparison. When we compared the coping strategies and styles by exercise level, we decided to include in the mean comparison analyses only the SA and AA subgroups due to the limited size of the LA subgroup. We found significant differences between the SA and AA groups in several coping endpoints. In particular, we found significant differences for one, Personal development and Problem solving). In all cases, active participants scored higher than their sedentary counterparts (see Table 3 and Figure 2).

Finally, we performed a hierarchical multiple linear regression analysis to determine the impact of coping styles and exercise during the pandemic on anxiety manifestations, controlling for educational level and pre-pandemic level of exercise. Sex-gender and age were introduced in a first analysis, but both were non-significant predictors and were omitted in further analyses to increase the statistical power of the test. The other sociodemographic variables were not included in the analysis due to the lack of representativeness of some of their levels. We found that only the Active method, the Avoidant method and exercise during the pandemic predicted participants’ anxiety manifestations (Table 4). For every 1 unit increase in these indicators, anxiety decreased 0.3 standard units (active method) or increased 0.2 standard units (avoidant method). Exercise positively predicted anxiety level (β = 0.15), which might point to that exercise is being used as a management strategy itself when the individual desires to deal with emotional distress. To test this new hypothesis, a similar hierarchical multiple linear regression analysis was conducted regressing exercise level based on coping styles and distress symptoms, controlling for pre-pandemic practice and education level (the same decisions on sociodemographic variables than for the previous analysis were adopted for the present one). Regular exercise habit, anxiety levels and Active coping style significantly predicted exercise levels. A 1-point increase in the predictors was associated with increases in exercise practice of 0.15 to 0.20 SD. Yet a marginally significant finding, Avoidant coping inversely predicted exercise (Table 4).

## 4. Discussion

The main objective of this study was to examine the current manifestations of anxiety and the coping strategies executed to manage emotional distress during an active stage of the COVID-19 pandemic in Spain in a group of adults of both genders with different sociodemographic and personal characteristics. Contrary to expectations (hypothesis 1), we found that average anxiety levels were 42 over 80, bordering on a mild anxiety problem. This might be due to the fact that an active method focused on managing the problem and dealing with emotional reactions was the most used coping style to handle the situation; and the coping strategies most used were more functional (e.g., personal development, positive reappraisal, planning, problem solving, acceptance) rather than dysfunctional ones (e.g., depressive reaction). Thus, all these coping resources might be helping individuals to successfully manage their emotional status. However, when dividing the sample by the anxiety index, it clearly appears that 32.5% have mild anxiety symptoms and 6.5% have moderate or severe anxiety symptoms, which means that more than 1/3 of the participants were suffering anxious symptomatology of noticeable intensity. Our results are in line with previous reviews and meta-analyses in which anxiety is considered an important consequence of living under the COVID-19 outbreak [3,4,11,12,13,14,15,16]. In addition, all these findings point to the importance of providing the community with the necessary coping skills for managing emotional distress. Thus, national and supranational organisms taking care of people’s health have the responsibility of developing widespread interventions focused on increasing the individuals’ and the communities’ resources for maintaining the mental health of worldwide citizens.

As we have emphasized, on average, the coping strategies and styles most used are mainly functional (hypothesis 2). However, the obtained scores for these skills are still moderate, and some other functional strategies that could be also helpful for the management of anxiety symptoms have not been profusely used (e.g., social support for problem solving, suppression of distracting activities). The strategies used by the participants are similar to the strategies found in previous research where the importance of behavioral and emotional coping is highlighted, since they have turned out to be the most beneficial [19,20,21,28]. Nonetheless, the creation of intervention programs aimed at training the most effective and helpful coping strategies would be advisable in order to improve people’s success when putting them into practice to deal with the emotional burden of a major life stressor such as a pandemic.

In this sense, physical activity can be viewed as a coping resource. Regarding active behavior, we found that the prevalence of a sedentary lifestyle is four times higher during the pandemic than in the pre-pandemic stage (21% pre-pandemic, 87% during the pandemic) (hypothesis 3). This worrying withdrawal during the pandemic is similar to that found by other authors [32,33,34,35,36,37,38,39,40,41]. The negative change respecting physical activity is due surely to the restrictions to prevent the virus spread (e.g., confinement, locking of outdoor and indoor facilities, social distancing), but also undoubtedly to motivational factors: practice in these conditions might be considered less fun or enjoyable, the lacking of appropriate home spaces or resources such as home-guided programs or the absence of knowledge or alternative resources. Consequently, both public and private entities are encouraged to educate people about the available exercise activities and resources for these extremely-limiting conditions.

Another aim of this study was to explore the relationship between anxiety, coping strategies and styles and levels of physical activity, as well as the impact of coping skills and exercise on participants’ manifestations of anxiety. As predicted (specific hypothesis 2.1), a number of coping strategies correlated with anxiety symptoms in the expected direction (i.e., more functional strategies—inverse association; maladaptive strategies—direct association). Similarly, two of the coping styles, the active and the avoidant methods, were also associated in the expected way with the manifestations of anxiety. Both methods accurately represent the use of functional (active method) and dysfunctional (avoidant method) coping, so these results are similar to those found in the reviewed literature [19,28,31].

Regarding physical activity, we found a relationship between exercise and the use of three functional coping strategies, namely Planning, Problem solving and Personal development, and an Active coping style (specific hypothesis 4.1). All of these associations may be indicating that people have used exercise during the pandemic along with other coping resources as a way to manage their mental and emotional states. These results highlight the importance of exercise as a coping strategy itself, and probably as a resource that is feeding from, and feeds, other adaptive coping strategies, since the use of functional coping strategies is related to a higher level of physical activity. Our results are similar to those found in several investigations [64,65,66,67,68,69]. Some other findings in the present research also support the coping role of exercise.

Specifically, we further compared the resources for coping with mental distress of the participants by their levels of physical activity during the COVID-19 pandemic. The results indicated differences between the sedentary and the active participants for Active method, and for Planning, Problem solving, Personal development and Acceptance strategies. In all cases, active participants scored higher than their sedentary counterparts (specific hypothesis 4.3). However, it is important to highlight that all the exercise-level groups more commonly used functional rather than maladaptive strategies to cope with distress. Even so, it should be noted that those participants who practiced higher levels of physical activity reached the highest scores. Yet most of the participants were physically active before the pandemic (≈ 80%) and thus may have internalized the association between coping strategies and physical activity as a protective measure for the management of mental distress, it is alarming the abandonment of such positive action in moments when it is more necessary. Following this rationale, it could be expected that previously active participants who are currently sedentary could stop using functional or more effective coping strategies, or start using more dysfunctional ones if the restrictions derived from COVID-19 are prolonged in time. Our results are also congruent with previous research where it is observed that a sedentary lifestyle can be associated to an increase in negative affect and emotional symptoms during the pandemic [48,49,50,64,65,66,67,68], as well as to the worsening of other healthy behaviors and of coping with distress [46,51]. In addition, the protective effect of previous practice of exercise has also been supported [59,60]. Perhaps, the mere fact of practicing exercise regularly (regardless of its features, such as intensity) makes the person resort to functional strategies based on his/her personal experience, giving this to exercise a differentiating role when it comes to knowing which coping strategies to choose [69].

Moreover, we also confirmed the combined impact of coping strategies and exercise on anxiety experiences. We found that an Active method inversely predicted participants’ anxiety manifestations, whereas an Avoidant method positively predicted it (specific hypothesis 2.2), but contrary to expectations (specific hypotheses 4.2 and 4.4), exercise during the pandemic was found to positively predict anxiety levels. The findings for exercise might seem counterintuitive, because it was expected an inverse association between exercise and anxiety, supporting the anxiolytic effect of physical activity [81,82,83]. Far from an apparent anxiety-induced effect of exercise, it is possible however that this pattern of association, along with the remaining of our findings as a whole, may be indicating that exercise is being used as a management strategy to struggle with distress and anxiety symptoms when they are experienced by the person (hypothesis 4). Supporting this, we found that regular exercise habit, anxiety levels and an Active coping style significantly predicted exercise levels, suggesting that physical activity is a coping strategy for dealing with emotional stress along with other active coping skills.

Complementarily, it is possible that the decrease in physical activity during the pandemic in previously regular practitioners induces higher levels of anxiety as a result of withdrawal, i.e., abstinence effect, as indicated by some studies and reviews [84,85] and recent reviews on studies carried out during the COVID-19 pandemic [86]. This is also congruent with all the research that positively associates lower levels of exercise and a sedentary lifestyle with higher levels of anxiety [87,88]. These findings reaffirm the beneficial impact of active, functional coping strategies on anxiety and the benefits of exercise on people’s mental health, as indicated in previous research [64,65,66,67,68].

Besides its contributions, this study has some limitations that deserve to be noted. The main limitation is the size and composition of the sample, with an overrepresentation of individuals who are female, in their young adulthood, highly educated and employed, making it necessary to confirm our results with larger and more heterogeneous, representative samples. Second, we have only considered total time of weekly practice to differentiate levels of active behavior, while many other parameters have been ignored (e.g., type, intensity or frequency of physical activity). Future research should include these characteristics as well as other aspects regarding reasons to practice and motives of habit changes. Additionally, it would be necessary to continue researching on the relationship between coping skills and exercise practice for managing psychological distress. All of this could help professionals to design and implement intervention programs tailored to the needs and expectations of the recipients. Only by knowing the association of coping skills with physical activity and their benefits, interventions can be developed that help people feel better and to cope more effectively with stressful events and distress. Finally, we have not taken into account the possible impact of some sociodemographic variables such as socioeconomic status, family composition, home conditions, etc. or even the personal experience with the COVID-19 disease. The empirical evidence indicates that their influence should be taken into account, and future investigation should be more rigorous when it comes to knowing the influence of these conditions as moderators or for controlling their effect as covariates. For the same reason, possible cultural influences should be explored. Therefore, to guarantee the generalizability of our findings beyond these limitations, research must be carried out in other nations and cultures and with citizens of different ethnicities.

Despite these limitations, our results highlight the convenience of addressing the implementation of functional coping strategies together with physical activity for promoting good mental health and alleviating mental distress. Our results also have important practical applications for program developers based on the importance of implementing adequate coping strategies, including active behavior, to minimize the possible harmful psychological effects (e.g., anxiety, stress, depression) produced by the pandemic and its consequences. Physical activity reduction or elimination in our daily routine can have negative effects, given the helpful effects of exercising, even at low levels [80,89,90]. Therefore, we highlight the need to implement interventions aimed at emphasizing the acquisition of functional coping strategies and training different exercise practices at home or outdoor in the general population, and particularly in people at risk or showing emotional symptoms, to promote mental health and prevent possible psychosocial difficulties during the times of a pandemic.

## 5. Conclusions

With a prevalence of anxiety symptoms of mild to severe intensity observed in >1/3 of the participants, functional coping strategies and active management styles are revealed as the most helpful for dealing with the emotional burden of the pandemic and its social and personal consequences. These coping skills are related also to a higher use of exercise as a coping strategy. We observed that regular exercisers used more frequently active coping and that exercise was associated with other forms of active, functional coping. In general, our findings supported that exercise was used as a coping strategy for dealing with emotional distress. Our results highlight the positive impact of the use of functional coping styles and of exercise practice for the management of negative states such as anxiety during the pandemic. They also underline the importance of developing interventions aimed at reducing emotional distress and optimizing physical and mental well-being of the population by means of promoting varied coping resources and decreasing the withdrawal from an active lifestyle.

## Figures and Tables

**Table 1:** Sociodemographic data from the participants.

Conditions		*N*	%
**Sex**	Women	140	70
	Men	60	30
**Age range**	Young adulthood: 18–44 yr.	170	85
	Middle and older adulthood: 45–74 yr.	30	15
**Marital status**	In a relationship/Married	32	16
	Single	136	68
	Separated/Divorced/Widow-er	32	16
**Education level**	Primary school	5	2.5
	High School	22	11
	University (current)	58	29
	University (finished)	94	47
	Postgraduate studies	21	10.5
**Employment situation**	Employed	61	30.5
	Studying	131	65.5
	Unemployed, including housework	6	3
	Retired, pensioner	2	1
**COVID-19**	Without symptoms/negative diagnosis	184	92
	Symptomatic but not diagnosed	4	2
	Positive diagnosis	12	6
**Number of cohabitants**	0–1	20	10
	2–3	131	65.5
	4 or more	49	24.5
**Number of children**	0	165	82.5
	1–2	28	14
	3 or more	7	3.5
**Pre-pandemic physical activity**	Sedentary	41	20.5
	Mild activity	44	22
	Active	115	57.5
**Physical activity during the pandemic**	Sedentary	173	86.5
	Mild activity	5	2.5
	Active	22	11

**Table 2:** Descriptive data and correlations between anxiety, coping strategies and styles and physical activity.

Variables (Possible Range of Scores)	*M*	*SD*	Anxiety *r*	Exercise *r*
Anxiety (20–80)	42.43	9.52	-	0.07
Coping strategies(0–12):				
1. Positive reappraisal	6.19	2.57	−0.29 **	0.10
2. Depressive reaction	4.81	2.01	0.07	−0.07
3. Denial	2.83	2.08	0.17 *	−0.09
4. Planning	6.07	2.40	−0.10	0.18 **
5. Acceptance	6.20	2.12	−0.23 **	0.14
6. Cognitive disconnection	4.84	2.35	0.15 *	−0.06
7. Personal development	7.56	2.53	−0.20 **	0.17 **
8. Emotional concealment	5.15	2.50	0.08	0.02
9. Emotional distancing	4.52	1.83	0.18 *	0.04
10. Suppression of distracting activities	4.18	1.92	−0.05	−0.02
11. Coping restrainment	5.37	2.20	0.01	0.06
12. Coping suppression	3.96	2.02	−0.03	−0.08
13. Problem solving	6.15	2.37	−0.18 *	0.16 *
14. Social support for problem solving	3.14	2.10	0.16 *	−0.01
15. Behavioral disconnection	3.30	1.85	0.05	−0.10
16. Emotional expression	5.76	2.38	0.08	0.12
17. Emotional social support	5.98	2.81	0.02	0.11
18. Palliative response	2.54	2.10	0.29 **	−0.07
**Coping styles:**				
Active method (0–72)	35.91	10.32	−0.18 *	0.17 *
Passive method (0–72)	30.65	7.82	−0.03	0.08
Avoidant method (0–72)	21.97	8.53	0.20 **	−0.09
Response-focused (0–72)	27.34	7.71	−0.05	0.02
Problem-focused (0–72)	29.69	7.66	−0.05	0.10
Emotion-focused (0–72)	31.51	7.80	0.11	0.13
Behavioral activity (0–108)	48.16	11.40	−0.05	0.09
Cognitive activity (0–108)	40.38	11.09	0.06	0.05

** *p* < 0.01; * *p* < 0.05.

**Table 3:** Descriptive findings and mean comparisons (Student’s *t* tests) for the coping strategies and styles by physical activity.

VARIABLES (Min–Max)	SA (86.5%)		LA (2.5%)		AA (11%)		SA vs. AA *t* (*p*)
	*M*	*SD*	*M*	*SD*	*M*	*SD*	
Positive reappraisal (0–12)	6.11	2.59	6.00	3.46	6.91	2.25	−1.382 (0.169)
Depressive reaction (0–12)	4.86	2.04	4.80	1.64	4.41	1.89	0.974 (0.331)
Denial (0–12)	2.90	2.08	3.00	2.12	2.27	2.10	1.323 (0.188)
Planning (0–12)	5.90	2.35	6.60	2.79	7.27	2.45	−2.561 (0.011 *)
Acceptance (0–12)	6.12	2.08	4.60	2.30	7.23	2.02	−2.366 (0.019 *)
Cognitive disconnection (0–12)	4.90	2.22	4.40	1.52	4.45	3.36	0.823 (0.412)
Personal development (0–12)	7.42	2.48	6.60	2.19	8.91	2.60	−2.633 (0.009 *)
Emotional concealment (0–12)	5.13	2.54	4.60	1.67	5.36	2.36	−0.405 (0.686)
Emotional distancing (0–12)	4.48	1.92	5.60	0.55	4.59	1.14	−0.266 (0.791)
Suppression of distracting activities (0–12)	4.20	1.92	3.00	1.73	4.23	2.00	−0.057 (0.954)
Coping restrainment (0–12)	5.34	2.16	4.40	2.19	5.86	2.51	−1.050 (0.295)
Coping suppression (0–12)	4.03	2.08	3.20	0.45	3.59	1.71	0.947 (0.345)
Problem solving (0–12)	6.01	2.39	6.00	2.83	7.27	1.83	−2.388 (0.018 *)
Social support for problem solving (0–12)	3.15	2.08	3.40	3.29	3.05	2.01	0.223 (0.824)
Behavioral disconnection (0–12)	3.36	1.82	3.80	2.17	2.68	1.94	1.631 (0.105)
Emotional expression (0–12)	5.64	2.37	6.60	4.16	6.50	1.92	−1.633 (0.104)
Emotional social support (0–12)	5.87	2.76	6.60	3.72	6.77	2.98	−1.435 (0.153)
Palliative response (0–12)	2.57	2.06	3.60	2.19	2.00	2.35	1.207 (0.229)
Active method (0–72)	35.29	10.19	34.80	15.58	41.09	8.97	−2.546 (0.012 *)
Passive method (0–72)	30.46	7.83	28.40	8.14	32.68	7.66	−1.256 (0.211)
Avoidant method (0–72)	22.23	8.44	23.60	5.32	19.59	9.71	1.359 (0.176)
Response-focused (0–72)	27.43	7.90	24.40	3.91	27.27	6.77	0.091 (0.927)
Problem-focused (0–72)	29.43	7.47	28.80	9.83	31.95	8.61	−1.464 (0.145)
Emotion-focused (0–72)	31.12	7.88	33.60	7.30	34.14	6.95	−1.715 (0.088)
Behavioral activity (0–108)	47.81	11.54	46.20	7.66	51.41	10.83	−1.388 (0.167)
Cognitive activity (0–108)	40.17	11.03	40.60	12.01	41.95	11.77	−0.708 (0.480)

* *p* < 0.05; SA: sedentary; LA: slightly active; AA: active.

**Table 4:** Significant predictors of anxiety considering active, passive and avoidant coping styles and the level of exercise during the pandemic (upper panel) and of exercise practice considering active, passive and avoidant coping styles and anxiety (lower panel) (with covariates, final models).

Variable		Predictor	Cor.R^2^	Stand. β	*t* (*p*)
Anxiety (*F* = 4.103, *p < 0*0.01)	Step 1	Education level	0.002	0.07	0.937 (0.350)
		Pre-pandemic exercise level		−0.07	−1.010 (0.314)
	Step 2	Active coping style	0.070	−0.31	−3.255 (0.001 **)
		Passive coping style		0.13	1.254 (0.211)
		Avoidant coping style		0.20	2.511 (0.013 **)
	Step 3	Current exercise level	0.086	0.15	2.072 (0.040 *)
Exercise during pandemic (*F* = 2.930, *p* < 0.05)	Step 1	Education level	0.016	−0.09	−1.239 (0.217)
		Pre-pandemic exercise level		0.14	1.972 (0.050 †)
	Step 2	Anxiety	0.019	0.15	2.072 (0.040 *)
	Step 3	Active coping style	0.055	0.20	2.030 (0.044 *)
		Passive coping style		0.02	0.160 (0.873)
		Avoidant coping style		−0.16	−1.938 (0.054 †)

** *p* < 0.01; * *p* < 0.05; † *p* < 0.10.
